# Collectanea

**Published:** 1833-09

**Authors:** 


### COLLECTANEA.

Floriferis ut apes in saltibus omnia libant,
Omnia nos, itidem, depascimur aurea dicta.

### PHYSIOLOGY.

### *Memoir on the Functions of the Encephalon, including a brief Notice of most of the recent Doctrines.*

M. Rolando considers that his experiments have proved that the medulla oblongata is the centre of sensibility, the focus and source of life. Treviranus regards it as the centre of animal life, at least. Fodera deems it to be the "hypomoclion" or under lever of life. It is, according to him, the main exciter of respiration, circulation, digestion, and of voluntary motion; the influence which it exerts, proceeding in an especial degree from the point whence the pneumogastric nerves arise; this point is the line of demarcation between the medulla oblongata and spinalis. The portion of the medulla situated below this point serves only to transmit impressions received; and the portion of the medulla oblongata situated above this point, is not the source of any influence, nor the centre of any action. The brain and cerebellum are only appendages to the medulla oblongata; and all irregularities of the sentient and moving powers which are observed in diseased states of the mass of the encephalon are referrible to sympathetic derangements in the functions of the medulla oblongata; for this latter part is invariably the only real and true seat of palsy. According to M. Serres, the whole encephalon is endowed with sensibility, but the medulla oblongata is the principal seat of it. M. Flourens places the source or centre of vitality, and of the nervous power, in the medulla oblongata.

All other parts of the nervous system, he says, are dependant on it. If the medulla be divided below the point at which the pneumogastric nerves arise, it dies, and the encephalic mass remains alive; if the section be made above this point, the very reverse ensues. Hence this point is termed by him the vital knot, the central bond of the nervous system, the base of the nervous tree, the encephalon representing the trunk, and the spinal cord and nerves, the roots of it. Respiration altogether depends primarily upon it; and it is not less indispensable for voluntary motion.

To none of these doctrines do we subscribe; the source and centre of life do not reside in, nor depend upon, any special organ, or set of organs, but each part is endued with vitality, and this vitality is a result of its organization. Neither animal nor organic life has an exclusive centre of influence or acting power; and, therefore, sensibility in its widest acceptation has no special, or particular seat. Every system, nay, every part of any, and more especially of the nervous system, has in fact an independent existence or vitality, and hence we cannot admit to any part exclusively what some authors have called "primordiality of action." Before we proceed further in our account of the functions of the medulla oblongata, let us determine what is the precise portion of the nervous system to which that term is to be applied. The older anatomists, and the most celebrated modern ones, include not only the rachidian bulb, or tail of the medulla oblongata, but also, the tuber annulare, and the crura cerebri et cerebelli; but we object to this classification; and, while we are inclined to restrict the appellation to the rachidian bulb, and the ganglions which it comprehends, viz. the pneumogastric, olivary or vocal, the acoustic or auditory, and the gustatory or trifacial ganglions, we annex to them the optic and olfactory bodies or lobules, which have usually been considered as belonging to the cerebrum. The reasons of our disjoining the tuber and the crura cerebri and cerebelli are that their organization, their mode of development, and their relative bulk, as well as the results of experiments upon them, do not agree with the structure, growth, or functions of the medulla; whereas, in these very particulars, there is a striking resemblance between the optic and olfactory lobules, on the one hand, and the rachidian bulb on the other; and we have therefore associated them together. The medulla oblongata, as thus defined, is, as it were, superadded to the spinal marrow, and the functions of the one are analogous to, but are more complicated and more perfect than those of the other; and again, it is subordinate, alike in organization, position, and complexity of function, to the other parts of the encephalic mass. The medulla oblongata is to the organs of special sense what the spinal cord is to the organs of general sensation and locomotion, (the skin and ordinary muscular apparatus.) The former, viz. the organs of the four special senses, are provided not only with ganglionic nerves (for the purposes of organic or nutritive life) and nerves of general sensation and of general muscular motion, but also with two sets of particular or superadded nerves; the one being destined to transmit peculiar impressions from the organ to the nervous centre; the other, to receive the motive or existative influence from this centre, and to convey it to the organ. Now with these nerves of special sensation are associated certain peculiar or proper motor nerves; thus the hypoglossal is associated with the lingual, the third and fourth pairs, the ophthalmic of Willis, and the 6th pair, with the optic; and the facial with the auditory. The sense of smell does not require any general movement, and its muscular apparatus or appendages are imperfect; it is therefore not provided with a special or proper motor nerve, but is supplied with twigs from the facial and trifacial nerves. The relation which we have thus pointed out to exist between the auditory and facial nerves, co-operating simultaneously for the same function, although in a different mode, and the consideration of the mutual relative development of these two nerves, shew us the error of those who have regarded them as quite unconnected with, and independent of each other. In the same manner, the pneumogastric nerve, on the one hand, and the spinal on the other, are in reality only two parts of the same nervous apparatus; the one consisting of sensitive filaments, and the other of motive filaments; co-operating with this apparatus, are other cords of filaments, sensitive as well as motor, which proceed from different parts; these cords are the glosso-pharyngeal, the hypoglossal, the phrenic, and the intercostals. The first phenomena, or set of actions, which follow any impression made on an organ of sense, and transmitted along its nerve to its respective ganglion, whether of the medulla oblongata, or spinalis, take place in the locomotive, or muscular apparatus with which the organ affected is more especially associated, certain movements occur, and for the purpose either of defending it from the too powerful impression of the external agent, or of better accommodating and arranging it, for such an impression; thus, in the case of the eye, or of the ear, the first movements which follow a noise, or strong light, are certain automatic and involuntary actions of the muscles subservient to the organs of vision, or of hearing: the object of which actions is either to mitigate, or to increase and concentrate the external impressions. Now these actions are under the influence solely of the medulla oblongata: but the perception of the impressions belongs to the brain, and to the brain alone; for the brain is the organ of the faculty which wills, and of the faculty which perceives and judges. We are therefore not to regard the medulla oblongata and spinalis as the seat of special and general sensation; they are, so to speak, points of transition, or organs intermediate between the external part on which the impression is made, and the brain, which perceives the impression: the function of the medulla being rather to excite a train of movements (those of general locomotion), destined to withdraw the individual organ, or to approximate it to the external agent, or, at other times, to modify and alter the condition of the organ itself; in short, to determine and regulate those special actions which are necessary to the accomplishment of the functions of sense.

MM. Majendie, Dumeril, and Serres, suppose that the trigeminal nerve is fundamentally the real nerve of all the special senses; that it bestows, by a mode of influence which we do not understand, the special sensibility on the optic, olfactory, auditory, and gustatory nerves; and that frequently, especially in the lower animals, it takes the place, and exercises the functions of these nerves. The following are the chief arguments adduced in favour of these opinions. 1. In the mole, there is no optic nerve, but, instead of it, there is a twig of the fifth pair; in fishes, there is no auditory nerve; in the cetacea, no olfactory nerve; and yet these animals have in all probability a certain degree of the corresponding sensations. 2. When the fifth pair is divided, all the special senses are destroyed, or at least enfeebled; when the special nerves alone are divided, the animal still retains in part the capability of perceiving odours, tastes, sounds, and light; but if the fifth pair is divided at the same time, these sensations are utterly and entirely annihilated. 3. Certain of the special sensations may be produced in other ways than the contact or impression of their ordinary stimuli; thus, for example, sound may be heard although the external ear be plugged up, if a watch be applied to the head, or put between the teeth. 4. The occurrence of amaurosis, anosmia, and ageustia, from neuralgia of the trigeminal nerve. In answer to these arguments, we may state that division of the fifth pair does not immediately and instanter destroy any of the special senses; that a certain interval elapses, and during this interval, certain changes have taken place in the texture of the parts on which the nerve was distributed; thus, in regard to the eye, the cornea becomes opaque, the conjunctiva inflamed, and the humours muddy; the same results follow any injury or disease affecting this nerve: very different, however, is the effect of the division of the optic nerve; the sight is gone instantaneously and for ever. As to hearing the ticking of a watch, when put between the teeth, it is easily explicable on the well-known laws which regulate the transmission of sonorous vibrations; in a similar manner can we understand that those animals which have no external ear, as seals, moles, fishes, &c., may yet have the sense of hearing. And again, can we not hear the noise of our own voices, even when the ears are firmly plugged, and excluded from any atmospheric inpulsions on the tympanum? We readily admit, that the fifth pair is intimately connected with the functions of the four special nerves; but this connexion is indirect, and arises from the trigeminal being the nerve of general sensibility, and of common mobility to the hand and face; and when it is destroyed, or disused, certain morbid changes take place in the organization of the structures which are dependent upon it. But let not our readers be deceived, and suppose that these organic changes which ensue, are a proof that the trigeminal nerve has any direct influence upon the circulation, absorption, exhalation, and secretions of the organs, either in a state of health, or of disease: all these functions, and all morbid states of them, such as inflammation, ulceration, œdema, &c. are dependent upon, and regulated by, the great sympathetic nerve, which we know to be connected by several twigs with the fifth pair. The only direct result of the injury of the fifth pair, is the loss of common sensibility in the exterior surface of the eye and face, palsy of the muscles of the face and tongue, and an immobility of the eye. It is curious that any lesion of the great sympathetic, even in the neck, is followed by inflammation, dulness, and atrophy of the eyes; in short, by all the consequences of an injury of the fifth pair itself.

Tiedemann observes that the great sympathetic, by the influence which it has on the general phenomenon of nutrition, and therefore on the organic condition of parts, and on the functions of these parts, such as the secretion of the humours of the eye, and of the mucus of the nose, must necessarily exert a great control over the phenomena of sensation, more especially of smelling and of sight. We have said above, that Majendie and others assert that the mole has no optic nerve; Carus and Geoffry St. Hilaire, however, are of a different opinion, and state that this animal has at least the orbitary portion of the nerve, that it is of the size of a hair, and is united to the ophthalmic branch of the fifth pair, to form with it the retina. This is still a disputed question; and, although Hilaire supposes that the functions of any of the sensorial nerves are never transposed to, nor performed vicariously by, another nerve or nerves, yet we cannot deny, that in the invertebral animals, and even in some families of the vertebral ones, the divisions of the trigeminal nerve appear to supply the place, and perform the duties of the proper nerves of sense. In those animals which have no olfactory or gustatory nerve, or in which they have been cut, the probability is, that the sensation which belong to these special nerves are reduced to the phenomena of ordinary and general feeling, or sensibility. There may be, quite possibly, a certain positive action of light on the minute and pulpy twigs of the fifth pair in the eye: for light must be regarded as a species of matter, and must therefore make an impression, however weak, and almost unappreciable, on a sentient part; but this impression is only a modification of the general phenomena of touch. The transformation, therefore, of nerves of general sensibility, into nerves of special sensation, is to be regarded as erroneous and unsatisfactory. Bell and Shaw have pointed out the many fallacies into which Majendie has been led, by confounding the phenomena of special, with those of common sensibility; and Eschricht has lately published a suite of experiments which are at utter variance with the conclusions of the French physiologist. —*Medico-Chirurgical Review.*

*Case of a Cyst containing a Human Fœtus, in the Mesentery of a Boy.* [From Baron DUPUYTREN's Lectures.]

THE visceral hydatids developed in the cavity of the pelvis may be confounded with a crowd of tumors too numerous to name, and which, moreover, have been repeatedly studied. But there is one especially deserving attention from its extreme curiosity, and which has been described in a periodical, now difficult to be procured, namely, the "Recueil des Mémoires de la Faculté de Médecine de Paris," and entitled case of a "cyst containing a human fœtus developed in the mesentery of a boy of fourteen."

"AMÉDÉE BISSIEU, son of M. Bissieu, of Vernueuil, department de l'Eure, was born in 1790, of a young, well-formed, healthy woman, who had previously borne another child, well-formed and of good constitution. On the night during which his mother supposes he was conceived, one of the alarms then so frequent in France threw the town into violent agitation, and called the inhabitants tumultuously to arms. During her pregnancy, Madame Bissieu experienced some mental afflictions, as well as frequent indispositions; nevertheless her labour was propitious. It was supposed that during labour an unusual quantity of water escaped through the vagina. Immediately after birth the infant was confided to a nurse, who finding him weakly and unhealthy, almost despaired of bringing him up. Returned to his father's house, he complained, from his first lisp, of pain in the left side of the chest and belly. The volume of these parts was so considerable that it was feared he laboured under organic disease; but the size was, nevertheless, so variable, that nothing was done beyond accommodating his clothes to these variations. However, as he grew up these fears subsided, but the boy's body continued thin; he continually complained of slight pains in the side; his appetite was fantastic and irregular, and he frequently suffered from indigestion. On dressing him one day it was perceived that two of his left ribs were more elevated and prominent than the others; but this was attributed to the effects of a habit he had contracted of sucking the right thumb, and inclining his body to that side. Still less attention was excited by the circumstance, as at this time the lad was distinguished for a degree of gaiety, vivacity, and intelligence, beyond his age. He was sent to a boarding-school at Rouen, where, having spent eighteen months, he was suddenly attacked, on the 13th Nivose, year twelve, with acute pain in the side and left hypochondrium, with continued fever, with exacerbations, and a feeling of oppression. Great swelling of the pelvic region also occurred. He was bled and purged. The fever continued, and the swelling made progress. On the seventh day of his illness, M. Blanche, the surgeon, perceived distinctly in the abdomen, a hard and very painful tumor extending along the false ribs to the crest of the ilium, rounded from side to side, and of the size of a large melon. Calming treatment was employed, but the pain did not diminish until an abundant purging of fœtid purulent matter had taken place. The marasmus, however, proceeded, and, after some months' useless treatment he was sent home. On his arrival, MM. Guerin and Bertin Desmardelles recognised the tumor. A continual cough soon occurred, accompanied by purulent and fœtid expectoration, and a purging of matter also fœtid, in the midst of which, six weeks before his death, was found a parcel of hairs rolled up on themselves. He died on the 23d Prairial, an. 12, six months after the attack at Rouen.

"*Autopsy.* The opening of the body took place next day, in the presence of MM. Guerin and Bertin Desmardelles. These physicians discovered in the left hypochondrium, below the spleen, a large, thick, membranous sac, adhering to all the surrounding parts, and especially to one of the large intestines, which they presumed to be the colon; and in this cyst, amidst purulent, thick, and yellow matter, two masses were of nearly equal volume, situated transversely before the vertebral column, one applied to the other, but, nevertheless, quite distinct. Of these two masses, one placed inferiorly, was composed of a large handful of tangled felted hair; around this were two little parcels of hair, like what passed by stool six weeks before death. The other mass, situated higher, consisted of an oblong, fleshy, and bony substance, covered with skin. At one of its extremities was seen an imperfect head, with hair, teeth, a deformed nose, a kind of orbit at one side, and an ear at the other. At the opposite extremity was a limb-like appendage, ending in some tongue-shaped points armed with nails. Lastly, from the centre of the mass proceeded a thick, short ligament, inserted into the parietes of the cyst."

"Deeming the case deserving of more minute researches, MM. Guerin and Desmardelles lifted the fleshy mass out of the pelvis, and took it away, together with the stomach, spleen, and a part of the large intestine. They ascertained, then, that there existed no trace of sexual conformation, external or internal, and that the sex of Amadée Bissieux was indisputably masculine. Lastly, they found, on dissecting the rest of the body, that the liver was very voluminous, and the lungs whitish and infiltrated with pus. Twenty-two days after this, the body was exhumed, in order to verify the facts now related. MM. Delzeuzes and Bronard, who were charged with the investigation, found no trace of any sexual organ but those belonging to the male. The bladder was cautiously separated, the vesiculæ seminales discovered; the rectum examined internally and externally, and nothing extraordinary found. Lastly, the external parts of generation were carefully inspected, and the testicles, vasa deferentia, and penis, were found to be perfect in formation, but small in size."

"*Remarks on the Case.* A fact so extraordinary excited universal attention, and M. Planche forwarded the preparation to the Faculty of Medicine of Paris, and I (said M. Dupuytren) was commissioned to report on this great anomaly in the laws of nature.

"The first fact I ascertained relative to the position of the fœtus, was, that it lay in a cyst of the transverse mesocolon, which had only communicated very recently with the intestine, through the destruction of a partition by which they were separated. In continuing this examination, I ascertained that the organized mass contained in the transverse mesocolon had many points of resemblance to a fœtus, but that it offered also numerous peculiar dispositions, some of which depended essentially on vices of conformation, while others appeared to be dependent on changes of form successively effected by time and the sojourn of the mass in the mesocolic cyst. The dissection of the mass was, however, the surest mode of determining the nature of this production. I did so with great care, and I discovered the trace of some organs of the senses, a brain, spinal chord, very voluminous nerves, muscles degenerated into a sort of fibrous matter, a skeleton, composed of a vertebral column, a head, a pelvis, and the rudiments of almost all the limbs. Lastly, in an umbilical chord, very short, and inserted into the transverse mesocolon, beyond the cavity of the intestine, an artery and vein ramified in each of their extremities in the fœtus, and the individual to which it belonged.

"The existence of these organs sufficed certainly to establish the individuality of this organized mass, although in other respects it was destitute of organs of digestion, of respiration, of the secretion of urine, and of generation. But the absence of these parts, at most, could only render it one of the monstrous fœtuses destined to perish at the moment of birth.

"We shall not dwell on suppositions, more or less problematical, advanced respecting the presence of this fœtus in the body of the young Bissieu. We shall only remark, that it is by no means rare to see twins born adhering by the back, belly, head, or by several parts at once. A degree of compression, more or less strong, exercised by the mother's organs on the soft embryos, either during conception or soon after it, may produce these monstrosities. In other cases, not extremely rare, the twins are so identified, that many organs are deficient in each, and are replaced by common organs, which serve for the existence of both individuals. In the first case, the montrosity depends on a mechanical cause, in the second it depends on a primary fault in the organization of the germs. One of these explanations being admitted, the sex of the individual who so long acted the mother to the fœtus in question, becomes altogether indifferent. The fœtus has progressed, but as extra-uterine conceptions do. In fact, to whatever part the fecundated germs are attached, their mode of nutrition is the same. They derive from all, by means of proper vessels, the nutritive fluids they require. They are developed, and increase in size, until the time ordained by nature for their expulsion, and if they cannot then be expelled, they putrefy and turn into adipocire; they dry, and become ossified, or else they vegetate until their presence, by irritating the adjoining parts, determines the formation of abscesses, and procures their discharge. Such is what seems to have happened in the case before us.

"To ascertain the degree of importance of this phenomenon, its exact cause should first be known, and then this importance would be determined by the light thrown on the natural process, and occasional irregularities of the process of generation. However, putting these considerations aside, the case does not the less merit our attention, from its rarity and interesting phenomena.—*Lancet.*

